# Supplementary material for: Risk to rely on soil carbon sequestration to offset global ruminant emissions
Source: Nat Commun. 2023 Nov 22;14:7625. doi: 10.1038/s41467-023-43452-3 (PMC10665458; doi:10.1038/s41467-023-43452-3)
Supplement: Supplementary file 1 — Supplementary Information [file 41467_2023_43452_MOESM1_ESM.pdf]

Supplementary Information

Risk to rely on soil carbon sequestration to offset global ruminant emissions

Yue Wang[1*], Imke J.M. de Boer[1], U. Martin Persson[2], Raimon Ripoll-Bosch[1], Christel Cederberg[2], Pierre J. Gerber[3,1], Pete Smith[4], Corina E. van Middelaar[1]

[1] Animal Production Systems group, Wageningen University & Research, P.O. Box 338, 6700 AH, Wageningen, the Netherlands

[2] Physical Resource Theory, Department of Space, Earth & Environment, Chalmers University of Technology, Sweden

[3] The World Bank Group, 1818 H Street NW, Washington, DC 20433, USA

[4] Institute of Biological and Environmental Sciences, University of Aberdeen, 23 St Machar Drive, Aberdeen, AB24 3UU, United Kingdom

Correspondence to: yue3.wang@wur.nl

Contents

Supplementary Note 1| Alternative scenarios to represent the long-term process of soil C-sequestration.

Supplementary Note 2| Determining cattle density based on manure N_2O and enteric CH_4 emissions.

Supplementary Note 3| Testing global aggregated results using MAGICC.

Supplementary Fig.1 | Global mean temperature change over 200 years as a result of (a total amount of 40 t) soil carbon sequestration under different assumptions on the timing of sequestration.

Supplementary Fig.2 | Number of cattle 'allowed' for a given (maximum) soil carbon (C) sequestration in grasslands to offset the climate impact of a possible range of manure N_2O emissions per cattle.

Supplementary Fig.3 | Number of cattle 'allowed' for a given (maximum) soil carbon (C) sequestration in grasslands to offset the climate impact of a possible range of enteric CH_4 emissions and manure N_2O emissions per cattle.

Supplementary Fig.4| Climate impacts of two scenarios using MAGICC climate model.

Supplementary Note 1| Alternative scenarios to represent the long-term process of soil C-sequestration.

In our approach, we translated long-term soil C-sequestration into a one-off pulse of CO_2 at year one to overcome data limitations and to facilitates the application of the approach. Supplementary Fig.1 shows the comparison in global mean temperature change between our assumption and three alternative pathways. The calculations were conducted based on a simple climate model [1] using a total sequestration of 40 t ha^{-1} as an example.

The following four scenarios were compared:

1) A 40 t C stock increase all happening in year one (this study);

2) Spread 40 t evenly over 100 years (i.e., 0.4 t C $year^{-1}$), assuming the soil constantly increases its C stock for 100 years. Within the century, the pathway of temperature change is different from our approach, whereas the result at year 100 (and hereafter) is relatively close;

3) Spread 40 t evenly over 20 years (i.e., 2 t C $year^{-1}$). Considering that in most cases, soil C stocks due to management changes (e.g., grazing) are expected to equilibrate over periods shorter than 100 years, and a 20-year time perspective that is widely used was adopted here [2-3]. Switching to 20-year of C-sequestration implies that, although the paths within the first few decades differ a bit, the differences between this assumption and our approach become negligible afterwards;

4) A 10 t C stock increase in year 1 with an annual declining rate of 25% and a total sequestration amount of 40 t over 100 years. This attempts to capture the fact that annual C-sequestration is not a constant value, and it is likely to reduce over years, i.e., the closer to the new equilibrium, the less it can sequestrate. This trajectory is assumed to be the most representative of the four scenarios. The result from this scenario is also very close to ours (especially after a few decades).

In conclusion, our result is largely consistent with other assumptions. Given the fact that the well-established global database on time-varying soil C-sequestration is not available or not sufficient, and the main point with the analysis is whether soil C-sequestration in grasslands is able to offset ruminant emissions in the long-term, which is not sensitive to the exact timing of the soil C stock changes, we believe our results are valid.

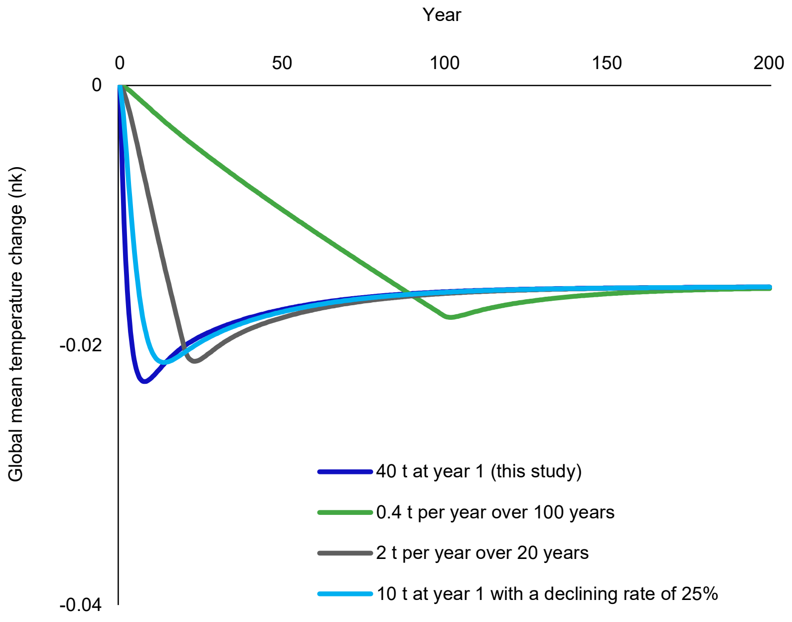

Supplementary Fig.1 | Global mean temperature change over 200 years as a result of (a total amount of 40 t) soil carbon sequestration under different assumptions on the timing of sequestration. The calculations were conducted based on a simple climate model [1]. Source data are provided as a Source Data file.

Supplementary Note 2| Determining cattle density based on manure N_2O and enteric CH_4 emissions

In the main body of this paper, we demonstrated how many cattle we could theoretically keep if aiming to completely offset enteric CH_4 emission over a 100-year period through soil C-sequestration (Fig.3). The same concept was applied to manure N_2O emissions (Supplementary Fig.2) as well as the combination of enteric CH_4 and manure N_2O emissions (Supplementary Fig.3). The results from both figures further support the conclusion that soil C-sequestration potential in grasslands can only possibly cancel out a continuous flow of CH_4 and/or N_2O emissions from ruminant production in very extensive systems.

Emission factors for N_2O emissions from manure management were calculated using the IPCC Tier 1 approach, which include direct and indirect N_2O emissions from the storage and treatment of manure before it is being used as fertilizer, fuel or others [2]. Based on the nitrogen excretion rate and animal weight of cattle in different world regions, the amount of nitrogen excreted by cattle was calculated to be 34-140 kg head^{-1} year^{-1}. Next, emission factors for direct and indirect N_2O emissions from excreted nitrogen in different manure management systems were derived from IPCC [2]. Direct N_2O emission factor varies from zero to 0.07 kg N_2O-N per kg of nitrogen excreted. Indirect N_2O emission factor varies from 0.0005 to 0.0072 kg N_2O-N per kg of nitrogen excreted and include those from the volatilization of ammonia and nitrogen oxide as well as from the leaching/runoff of nitrate. By combining the above values, we calculated that the N_2O emissions from manure management range from 0.04 (lowest level of excretion, Latin America, manure system of daily spread) to 16 (highest level of excretion, North America, manure system of cattle and swine deep bedding) kg per cattle per year. Note that the N_2O emissions that arise from the manure applied to land after treatment or directly deposited by grazing animals were reported separately by IPCC, whereas when assuming all manure is deposited on grasslands during grazing resulted in an N_2O emission factor of 0.6-1.9 kg N_2O per head^{-1} year^{-1}, which falls in the range of values applied in this study. For the purpose of display, we rounded the range of N_2O emission factor into 1 - 16 kg head^{-1} year^{-1}.

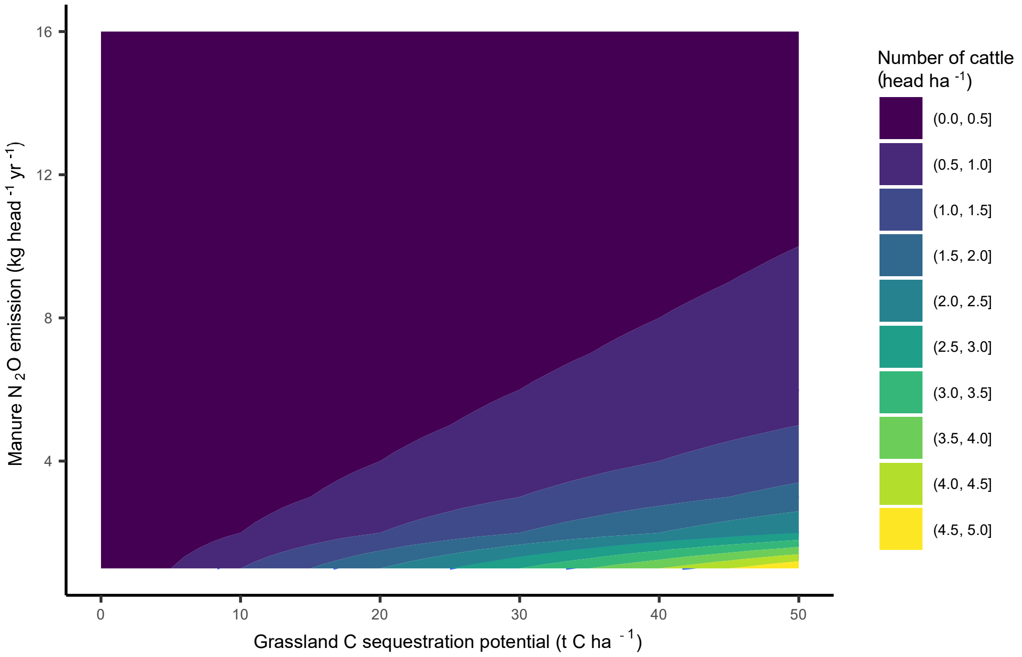

Supplementary Fig.2 | Number of cattle 'allowed' for a given (maximum) soil carbon (C) sequestration in grasslands to offset the climate impact of a possible range of manure N_2O emissions per cattle. Manure N_2O emissions include direct and indirect N_2O emissions from the storage and treatment of cattle manure. The values represent a wide range of cattle types, regions and manure systems. It was assumed that one tonne (t) of C would offset a continuous emission of 0.1 kg N_2O per year over 100 years, based on a simple climate model [1]. Source data are provided as a Source Data file.

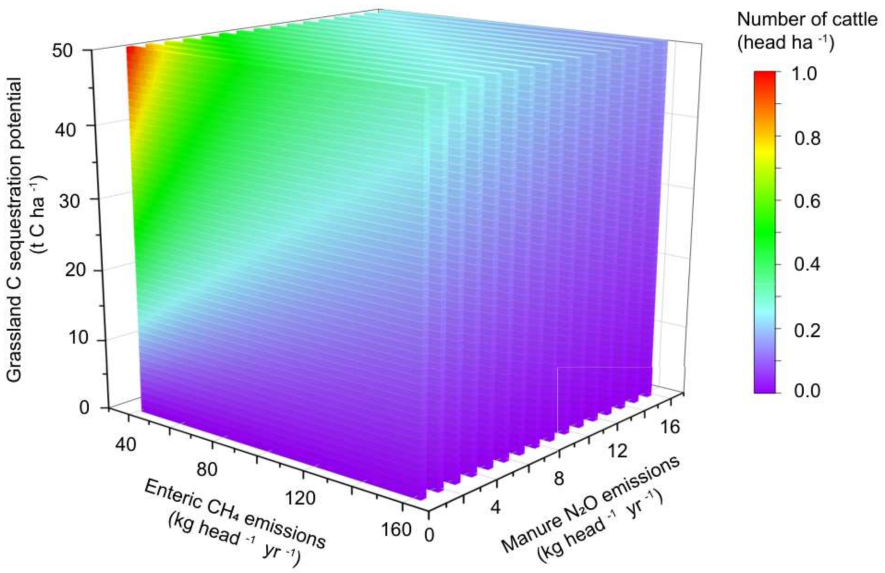

Supplementary Fig.3 | Number of cattle 'allowed' for a given (maximum) soil carbon (C) sequestration in grasslands to offset the climate impact of a possible range of enteric CH_4 emissions and manure N_2O emissions per cattle. The values for enteric CH_4 emission and manure N_2O emissions represent a wide range of cattle types, including productivity, animal age, breed, size, diet, production systems, manure systems and so on. It was assumed that one tonne of C would offset a continuous emission of 0.99 kg CH_4 or 0.1 kg N_2O per year over 100 years, based on a simple climate model [1]. Source data are provided as a Source Data file.

Supplementary Note 3| Testing global aggregated results using MAGICC

Given that the simple linearized climate model used in this study was made for small perturbations, we tested our global results using another climate model, i.e., Model for the Assessment of Greenhouse Gas Induced Climate Change (MAGICC). It is a reduced-complexity climate model that captures some of the bio-geophysical non-linearities in the climate system [4-5]. The online tool of MAGICC7 was used and could be found via https://live.magicc.org/. Among five main shared socioeconomic pathways (SSPs) scenarios that represents different future socio-economic projection and political environment (i.e., SSP1-1.9, SSP1-2.6, SSP2-4.5, SSP3-7.0 and SSP5-8.5), SSP2-4.5, a middle-of-the-road scenario, was selected as a baseline of our analysis. SSP2-4.5 indicates that the trends broadly follow their historical patterns and the nominal radiative forcing level by 2100 is 4.5 W m^{-2}.

Based on our global aggregated results (i.e., to offset the annually emissions of 110 megatons (Mt) of CH_4 and 2.4 Mt of N_2O, we need to increase SOC stock in global grassland by 135 gigatonnes (Gt) of C), two scenarios were developed and run by MAGICC7. Scenario 1 assumed a sequestration of 135 Gt of C (495 Gt of CO_2) from year 2020 to 2040, and scenario 2 assumed that annual emission of 110 Mt CH_4 and 2.4 Mt N_2O from year 2020 to 2100 were avoided. Their climate impacts were compared in terms of radiative forcing and global mean temperature change, and the differences could be seen in Supplementary Fig.4. Although the temporal paths of the two scenarios slightly diverge, it is believed that the differences will not challenge our main conclusion and that results confirm that it is not feasible to solely rely on soil C-sequestration in grasslands to offset warming effect of emissions from current ruminant systems.

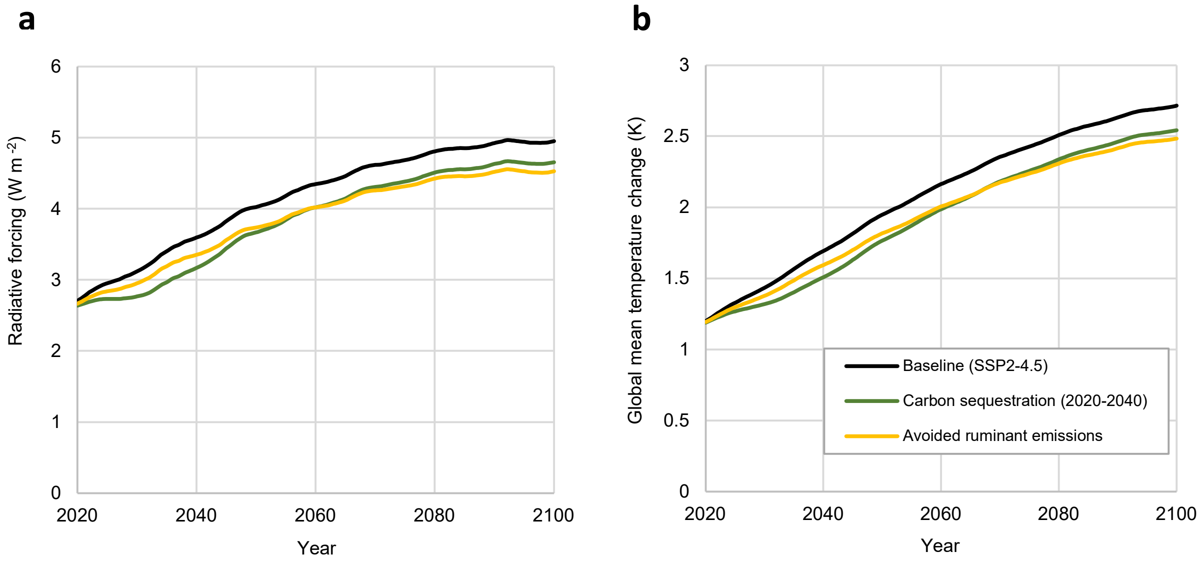

Supplementary Fig.4| Climate impacts of two scenarios using MAGICC climate model. The scenarios were developed based on the global aggregated results of this study, with scenario 1 assumed a sequestration of 135 Gt of C from year 2020 to 2040, and scenario 2 assumed that annual emission of 110 Mt CH_4 and 2.4 Mt N_2O from year 2020 to 2100 were avoided. The scenarios were run and compared by the Model for the Assessment of Greenhouse Gas Induced Climate Change (MAGICC), and the shared socioeconomic pathways (SSP2-4.5), a middle-of-the-road scenario, was selected as a baseline [4-5]. Source data are provided as a Source Data file.

Supplementary References

1. Persson, U. M. & Johansson, D. J. A. Simple climate model (Version 2.0). doi:10.5281/ZENODO.5957222. (2022).
2. IPCC. 2019 Refinement to the 2006 IPCC Guidelines for National Greenhouse Gas Inventories, Calvo Buendia, E., Tanabe, K., Kranjc, A., Baasansuren, J., Fukuda, M., Ngarize, S., Osako, A., Pyrozhenko, Y., Shermanau, P. and Federici, S. (eds). Published: IPCC, Switzerland (2019).
3. Smith, P. Do grasslands act as a perpetual sink for carbon? Glob Chang Biol **20**, 2708–2711 (2014).
4. Meinshausen, M., Raper, S. C. B., & Wigley, T. M. L. Emulating coupled atmosphere-ocean and carbon cycle models with a simpler model, MAGICC6 – Part 1: Model description and calibration, Atmos. Chem. Phys., **11**, 1417–1456 (2011).
5. Meinshausen et al. The shared socio-economic pathway (SSP) greenhouse gas concentrations and their extensions to 2500. Geosci. Model. Dev **13**, 3571-3605 (2020).
